# Notes and News

**Published:** 1896-02-15

**Authors:** 


### NOTES AND NEWS.

(COLLECTED AND COMMUNICATED.)

THE annual general meeting of the governors of the London Fever Hospital was held on Friday, 14th inst., at a quarter past five p.m., at the Hotel Victoria, Northumberland Avenue, W.C.

THE Right Hon. Lord Charles Bruce has consented to preside at the festival dinner of the Royal Hospital for Diseases of the Chest, City Road, E.C., which is to take place at the Hotel Metropole on Tuesday, May 26th next.

THE Rev. R. Bamford has been elected hon. secretary of the Yeatman Hospital at Sherbourne in place of General Waller, who has retired. General Waller will be greatly missed at the hospital, to which he has given his most valuable services during thirteen years. Many of the improvements in the administration, and especially in the comfort of the nurses, are due to his energy.

IT is hoped that the Duke of York will accept the invitation of the Mayor of Halifax and the president of the infirmary to open the new institution. Sir Savile Crossley has accepted the office of president for the next three years. Sir Savile Crossley takes an active interest in hospital matters, and his generosity has been amply testified, especially in relation to the Metropolitan Hospital Sunday Fund.

A CONTEMPORARY medical journal, in describing the depressing form of ailment which it designates "house nerves," remarks that in Russia "tea and Tolstoi" are guilty agents, whilst in America the negro question, iced drinks, candy, and business hurry help the complaint. Outdoor exercise and innocent amusement are prescribed as the panacea, but, as our contemporary remarks, it is not easy to apply a remedy to persons who have already contracted a morbid attachment to the evil causes of their nervous condition.

THE quarterly court of the governors of the Victoria Hospital for Children will be held in the board-room of the hospital on Wednesday, February 19th, at five o'clock.

A GOOD deal of sickness has been recently reported from Ashanti. Nevertheless, a French medical paper draws attention to the superior health and hygienic arrangements of the Ashanti expedition as compared with the French expedition to Madagascar.

THE annual report of the City of London Truss Society shows that 10,263 instruments were supplied to patients. Whilst the work of the society has largely increased during the present year, the expenditure has increased also through necessary improvements to the building being effected. Subscriptions are urgently needed.

THE Cumberland Infirmary will benefit to the extent of about £400 per annum through a trust fund amounting to £20,000, which is to be devoted to the support of the infirmary and to the maintenance of two district nurses for the town of Penrith and surrounding localities. The gift to the infirmary is attended with the stipulation that six beds shall be reserved for the use of patients from Penrith and certain districts around it, and that an ambulance shall be maintained for the removal of patients from the station.

THE ceremony of re-opening two wards at St. Thomas's Hospital by H.R.H. the Duke of Connaught will take place on the 21st inst. Mr. J. Wainwright, the treasurer, writes in this connection: "I beg you to accept my best thanks for your past support, and I trust you will once again impress upon the public the necessity of supporting by annual subscriptions this hospital, which is endeavouring to provide in the most economical way some of the 1,343 beds so urgently needed in South London."

WE regret to announce the death of Mr. Archie T. Collum, M.B., F.R.C.S., assistant surgeon to Charing Cross Hospital. He died on February 11th, at the hospital, where he has been as a patient since February 6th. His career had been a distinguished one. He was demonstrator of anatomy and surgical tutor in the medical school, and had held most of the resident appointments in the hospital, was a member of several of the medical societies, and had contributed various articles to the Journals and Transactions.

A MOST retrograde petition is now before the Lower Austrian Government. This petition pleads for the establishment of a medical school for "cuppers," or peasant doctors. The presence of these unskilled persons in Upper Austria and the Tyrol has made it practically impossible for medical men to find a living there. The charge made by the peasant doctor is about twopence a visit, and he is infinitely preferred to the qualified physician by the country people, whose prejudices are most difficult to surmount. It is said that no medical man has yet protested against the petition, which aims at placing the charlatan on a firm basis in Austria. The author of the petition, which the Provincial Council of Upper Austria has put forward, is a lawyer and a graduate of the University of Vienna.

